# Key Factors Determining the Success of Cardiopulmonary Resuscitation: A Multi-center Study Investigating Survival Rates and Predictors

**DOI:** 10.7759/cureus.78236

**Published:** 2025-01-30

**Authors:** Jamil M Baljoon, Jafar N Jamjoom, Khalid O Alolasi, Baraa S Tabbakh, Abdulhakim M Badawi, Mohammed K Almazmumi, Ibrahim Qasim Alanazie, Ahmed Hussein Alkohlani, Zainab A Alsaleh, Sawsan Hanafi

**Affiliations:** 1 College of Medicine, King Saud Bin Abdulaziz University for Health Sciences, Jeddah, SAU; 2 Research Department, King Abdullah International Medical Research Center, Jeddah, SAU; 3 College of Medicine, King Saud Bin Abdulaziz University for Health Sciences, Riyadh, SAU; 4 Research Department, King Abdullah International Medical Research Center, Riyadh, SAU; 5 College of Nursing, King Saud Bin Abdulaziz University for Health Sciences, Al-Ahsa, SAU; 6 Research Department, King Abdullah International Medical Research Center, Al-Ahsa, SAU; 7 Emergency Medicine Department, King Abdulaziz Medical City, Jeddah, SAU

**Keywords:** ambulance, cardiac arrest, cardiopulmonary resuscitation, cpr, duration of cpr

## Abstract

Introduction

Cardiac arrest can be fatal if not addressed quickly, and cardiopulmonary resuscitation (CPR) is a critical intervention to sustain life in cases of cardiac arrest. Multiple factors affect the mortality of patients who had cardiac arrest and subsequently CPR, such as the timing at which CPR is started. Due to the scarcity of local evidence regarding cardiac arrest in Saudi Arabia, this study aims to identify the predictors of CPR success and survival rate, namely, return of spontaneous circulation (ROSC). Factors that will be investigated are gender, timing, initial rhythm, and whether the cardiac arrest was witnessed.

Methods

A multicenter retrospective cohort study was conducted on patients who had cardiac arrest and CPR in National Guard Health Affairs in Saudi Arabia. The study was conducted in the Riyadh, Jeddah, and Al-Ahsa regions. The study's total sample was 949 patients, as 857 patients were from Riyadh, 58 patients were from Jeddah, and 34 patients were from Al-Ahsa. Furthermore, the sampling technique was a consecutive non-random sampling technique.

Results

Of the 949 cases of CPR for patients with cardiac arrest, 544 of 949 (57.3%) achieved ROSC after the first CPR attempt. Statistically speaking, female patients were more likely to have ROSC than male patients, with a percentage of 25.7% versus 19.1%, respectively (p = 0.0231). Also, patients who had ROSC had a mean duration before starting CPR of 4.95 minutes. However, patients who passed away had a mean duration before initiating CPR of 19.81 minutes (p < 0.0001).

Conclusion

Statistical analysis revealed that multiple factors can affect the mortality of patients who had cardiac arrest, one of which is gender, duration before initiating CPR, whether the cardiac arrest was witnessed, and the initial rhythm of the cardiac arrest. However, further local and international prospective designs are essential to study the variable factors that can affect the success rate of CPR, mainly ROSC. One of the factors that could not be measured in this study is cultural differences in response times.

## Introduction

According to the American Heart Association (AHA), cardiac arrest is defined as the sudden loss of the heart's function [1]. Cardiac arrest is caused when there is a malfunction in the heart's electrical system, which causes the person's heart to stop pumping [1]. Sudden cardiac arrest can be fatal if not addressed quickly [2]. In other research, it was shown that delay in the initiation of cardiopulmonary resuscitation (CPR) caused a decrease in survival rates [2]. CPR, the use of a defibrillator, or even simple chest compressions can increase the odds of survival until help arrives, and with adequate medical attention, survival is feasible [1]. In the United States, 200,000 episodes of in-hospital cardiac arrest and 356,000 episodes of cardiac arrest are documented each year [1,3]. According to a meta-analysis regarding the survival rate of in-hospital CPR, the immediate survival rate is 40.7%, and the survival-to-discharge rate is 13.4% [4]. A local study regarding in-hospital CPR has also proven that the initial success rate of CPR does not equal the survival-to-discharge rate. The estimated survival rate for immediate resuscitation is 64%, yet the survival-to-discharge rate is 30% [5]. Multiple factors affect CPR's success, such as the timing at which CPR is started [1]. According to the AHA, if advanced life support is initiated within three to five minutes following cardiac arrest due to ventricular fibrillation, the survival rate increases by 50% [1]. However, for every minute that defibrillation is delayed, the likelihood of recovery is reduced by 7-10% [6].

Regarding age and gender, a study done in the hospital of Kermanshah in 2013 revealed that the age and sex of the patients had no statistically significant link with CPR success rate [7]. On the other hand, a study by the AHA showed that females and those younger were likely to have longer durations of resuscitation. One possible explanation is that the health care providers have a perceived notion that these patients will benefit from more extended trial CPR. Even though some data suggest that there are no differences in demographics, other data from different literature stated that patients who were male, older, non-white, and admitted from a skilled care facility had a reduced chance of survival [8-9]. In summary, age, gender, race, cause of the event, comorbidities, location of arrest, and hospital factors all affect the outcomes of CPR. Since evidence is scarce regarding factors that affect the survival rate of CPR in the National Guard Health Affairs (NGHA), it is important to conduct this study to acknowledge the factors that affect survival, which may lead to a change in hospital practices if there are variables that significantly affect the success rate of CPR. The aim and significance of this study are to evaluate the risk factors in patients who experienced cardiac arrests to have better outcomes and a greater survival rate. Also, the result of this study could help determine future guidelines and protocols.

## Materials and methods

Ethical approval

This study was approved by the King Abdullah International Medical Research Center (KAIMRC) Institutional Review Board (study number: NRJ22J/156/05). Since all patients' information was kept confidential, no explicit consent from patients was necessary for this study.

Design and setting

This is a multi-center retrospective cohort study that investigated the medical records of patients who had at least one episode of cardiac arrest in NGHA hospitals in Jeddah, Riyadh, and Al-Ahsa in Saudi Arabia in the period between April 2017 and August 2022. The investigators opted for a retrospective study since there is a large number of prior patients that have not been investigated in the past. This study's missing data resulted from the study design since some healthcare workers may not have documented the full details of the cardiac arrest cases.

The sampling technique was a non-probability consecutive sampling technique with a total number of 949 patients. The data on cardiac arrest cases was collected using the hospital information system in NGHA (BestCare).

The qualitative variables measured in this study were age, gender, the state of the cardiac arrest, the initial rhythm of the patient during cardiac arrest and whether the rhythm has changed, the final rhythm after the cardiac arrest, whether the patient required intubation, complications of the cardiac arrest, and the outcome of the cardiac arrest. The initial rhythm in this study is defined as the first rhythm that appeared in the electrocardiogram (ECG) prior to or during the CPR process. The final rhythm in this study is defined as the rhythm that is observed in the ECG following the completion of CPR. Also, return of spontaneous circulation (ROSC) is the immediate pulse return in patients after the cardiac arrest intervention. In addition, shockable rhythm in this study includes ventricular tachycardia and ventricular fibrillation.

Furthermore, the quantitative data sets measured in this study were the durations related to the CPR, such as the duration before starting CPR, the duration of ambulance response to home, the duration till the ambulance arrived at the emergency department (ED), the total duration of CPR, and the number of shocks given to patients.

The inclusion criteria include patients with a documented code blue episode in NGHA hospital. The exclusion criteria consist of pediatric patients aged <18 years old, patients who signed a Do Not Resuscitate (DNR) form, and patients who did not have an episode of cardiac arrest (falsely reported code blue). Falsely reported code blue involves patients who seem unconscious but still have a pulse, thus indicating a state other than a cardiac arrest.

Data analysis

This study used J. Macintosh Project (JMP) Pro 17.0.0 (SAS Institute Inc., Cary, USA) for statistical analysis. The categorical data included age, gender, cardiac arrest state, state of intubation, cardiac arrest complication, cardiac arrest outcome, initial rhythm, change in rhythm, and final rhythm, and they were depicted by percentages and frequencies. The numerical data in this study included duration before CPR, duration till the ambulance arrived home, duration till the ambulance arrived at the ED, and the number of shocks; mean and standard deviation (SD) were used to depict the data.

The significance of the qualitative-to-qualitative data was depicted by chi-square. The independent T-test depicted the significance of the parametric qualitative-to-quantitative inferential data, and the significance of the non-parametric qualitative-to-quantitative data was measured using the Mann-Whitney U test. P-values less than 0.05 were considered statistically significant.

## Results

Demographics of the patients

Among the 949 cases of cardiac arrest, 672 (70.8%) were male patients, and 277 (29.2%) were female patients. Also, 857 (90.3%) cases were documented from the Riyadh region, 58 (6.1%) cases were from Jeddah city, and 34 (3.6%) were from the Al-Ahsa region.

Of the 949 cardiac arrest cases, 680 (71.7%) were unwitnessed cases of arrest, 246 (25.9%) were cases witnessed in the ED, and 23 (2.4%) were cases witnessed by paramedics. The aforementioned data is shown in Table 1.

**Table 1:** Demographics of the patients

	Number (N)	Percentage (%)
Total number of patients in the study	949	100
Gender of affected patients		
Male patients	672	70.8
Female patients	277	29.2
Setting of cardiac arrest		
Witnessed cardiac arrests in the emergency department	246	25.9
Witnessed cardiac arrests by paramedics	23	2.4
Unwitnessed cardiac arrests	680	71.7
Data collection source (in number of cases)		
Riyadh Hospital	857	90.3
Jeddah Hospital	58	6.1
Al-Ahsa Hospital	34	3.6

Duration of cardiopulmonary resuscitation

Only 440 of the 949 cases had clear documentation of time before initiating CPR. The mean number of minutes before initiating CPR was 16.93 (95% CI 14.9-18.96), as in Table 2.

**Table 2:** Measurements of mean, confidence interval, and standard deviation for different parameters CI: confidence interval; N: number of patients; CPR: cardiopulmonary resuscitation

Parameter	Mean	95% CI	Standard deviation
Before initiating CPR (N = 440)	16.93	14.9-18.96	21.67
Minutes stayed in the ambulance (N = 140)	18.15	16.56-19.73	9.48
Minutes till ambulance arrival at home (N = 103)	19.32	16.18-22.46	16.05
Total duration of CPR (N = 809)	21.59	20.45-22.74	16.56

Of the 440 cases, 42 (9.6%) cases had CPR initiated within less than 1 minute; 126 (28.6%) cases had CPR initiated within 1 minute; 24 (5.5%) had CPR initiated between 2 and 9 minutes; 55 (12.5%) had CPR initiated within 10 minutes; 39 (8.9%) had CPR initiated within 11-19 minutes; 37 (8.4%) had CPR initiated within 20 minutes; 7 (1.6%) had CPR initiated within 21-25 minutes; 49 (11.1%) had CPR initiated within 26-30 minutes; and 60 (13.6%) had CPR initiated within more than 30 minutes.

There were 140 cases that were brought by ambulance to the ED. The mean number of minutes for staying in the ambulance till reaching the ED was 18.15 minutes (95% CI 16.56-19.73). Of the 140 cases, most patients stayed in the ambulance for 10 minutes and 11-19 minutes, with that occurring 31 (22.1%) and 36 (25.7%) times, respectively.

Among the 140 cases brought by an ambulance, 103 had clear documentation of the number of minutes until the ambulance arrived at the patient’s home. The mean number of minutes for the ambulance to arrive was 19.32 (95% CI 16.18-22.46).

**Figure 1:**
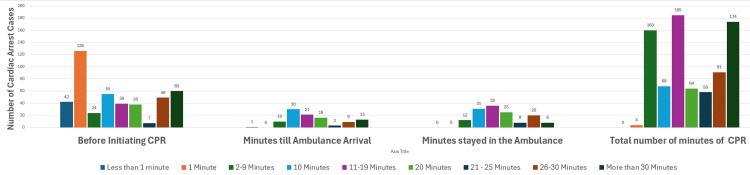
Frequency of different parameters distributed by number of minutes CPR: cardiopulmonary resuscitation

Of the 103 cases, 1 (0.7%) ambulance arrived home within less than 1 minute; 10 (9.7%) arrived within 2-9 minutes; 30 (29.1%) arrived within 10 minutes; 21 (20.4%) arrived within 11-19 minutes; 16 (15.5%) arrived within 20 minutes; 3 (2.9%) arrived within 21-25 minutes; 9 (8.7%) arrived in 26-30 minutes; and 13 (12.6%) arrived in more than 30 minutes, as illustrated in Figure 1.

From the 949 cases of CPR documented in this study, 806 had clear documentation of the total duration of CPR. The mean number was 21.59 (95% CI 20.45-22.74).

Rhythm in cardiopulmonary resuscitation

**Figure 2:**
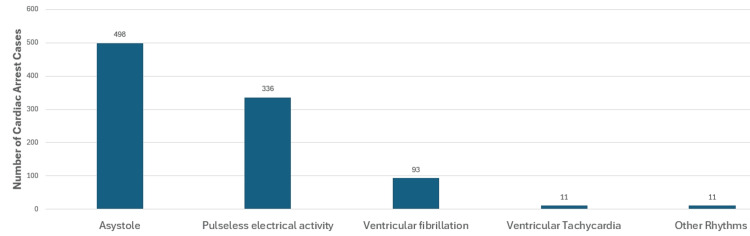
Initial rhythm before starting cardiopulmonary resuscitation (CPR)

The initial rhythm before doing CPR was documented in the 949 cases in this study, 498 (52.5%) of which were asystole rhythm; 336 (35.4%) were pulseless electrical activity; 93 (9.8%) were ventricular fibrillation; 11 (1.6%) were ventricular tachycardia; and 11 (1.6%) were other types of rhythms, as depicted in Figure 2.

Many patients had a change in rhythm during the process of CPR. 460 (48.5%) patients did not have a change of rhythm. The most common rhythm changes during CPR were pulseless electrical activity (PEA), occurring 120 (12.7%) times; ventricular fibrillation, occurring 114 (12%) times; and asystole, occurring 111 (11.7%) times.

**Figure 3:**
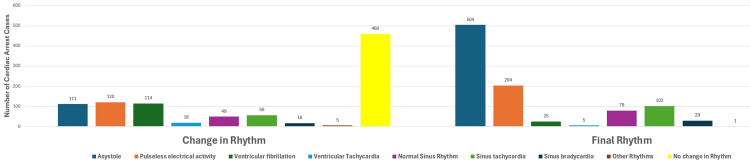
Frequency of change in rhythm during cardiopulmonary resuscitation (CPR) and the final rhythm after ending CPR

In regards to the final rhythm at the end of CPR, 504 of the 949 patients (53.1%) had asystole; 204 (21.5%) had PEA; 102 (10.8%) had sinus tachycardia; 79 (8.3%) were normal sinus rhythm; 29 (3.1%) had sinus bradycardia; 25 (2.6%) had ventricular fibrillation; 5 (0.5%) had ventricular tachycardia; and one patient had unclear documentation, as illustrated in Figure 3.

Of the 949 cases of CPR in this study, 171 (18%) had a shockable rhythm while doing CPR. For the 171 patients that had a shockable rhythm, synchronized cardioversion was utilized 12 (7%) times, and defibrillation was utilized 159 (93%) times to control the rhythm. The mean number of shocks required to control the rhythm was 3.63 (95% CI 3.11-4.15). Fifty-eight (33.9%) cases required one shock to control the rhythm, 25 (14.6%) cases required two shocks, 22 (12.9%) required three shocks, 20 (11.7%) required four shocks, and 46 (26.9%) required five shocks or more.

Procedures during cardiopulmonary resuscitation

There were 745 cases of CPR with documentation about intubation. Three hundred forty-one (45.8%) cases did not require intubation, 302 (40.5%) required intubation, and 102 (13.7%) cases were already intubated before initiating CPR.

Also, 119 of the 949 cases had difficulty obtaining a peripheral line. Hence, intraosseous cannulation was performed in 66 (55.5%) cases; a central line was inserted in 42 (35.3%) cases; and the remaining 11 (9.2%) had multiple procedures.

Complications of cardiopulmonary resuscitation

There were three main complications during the CPR. The most common notable complication during CPR was failure to do IV cannulation, which was observed in 49 of 949 (5.2%) cases. The second most common complication of CPR was failed intubation, which occurred in 18 of 949 cases (1.9%). Another complication that was a direct result of CPR was rib fracture, which was documented in six cases.

In some cases of cardiac arrest, there were factors that disturbed the CPR process. For example, family disturbance and overcrowding were reported in 10 different cases. Also, late arrival of staff was reported in three cases. Similarly, dysfunctional equipment was reported in three cases.

Outcome of cardiopulmonary resuscitation

Five hundred forty-four cases (57.3%) achieved a ROSC after the first CPR process. However, 131 (24.1%) had at least one cardiac arrest before discharge. Of the 131 cases that had at least one cardiac arrest before discharge, 73 (55.7%) had one more case of cardiac arrest; 31 (23.7%) had two more cases of cardiac arrest; 18 (13.7%) had three more cases of cardiac arrest; 4 (3.1%) had four more cases of cardiac arrest; and 5 (3.8%) had five or more cases of cardiac arrest.

Predictors of the outcome

The outcome of the cardiac arrest was well documented in 946 patients since there were three cases in this study with improper documentation of the outcome. There was a significant difference between the outcome of CPR and the gender factor since 128 out of the 542 male patients (19.1%) with documented outcomes had been discharged alive. On the other hand, 71 of the 205 (25.7%) female patients with documented outcomes had been discharged alive (p = 0.0231).

There was a significant difference between the time taken to initiate CPR and the outcome of CPR. As mentioned previously, the period before starting CPR was documented in 438 patients. The mean number of minutes before initiating CPR in patients who were discharged alive was 4.95 (95% CI 0.47-9.44), whereas the mean number of minutes before initiating CPR for patients who passed away was 19.81 (95% CI 17.63-21.99).

There was a significant difference between the CPR outcome and the total CPR duration. The total duration was clearly documented in 806 patients, of whom 168 were discharged alive, with a mean duration of CPR of 12.86 minutes (95% CI 10.44-15.29). On the other hand, the mean duration of CPR in minutes for those who passed away was 23.9 (95% CI 22.65-25.14).

There was a significant difference between the number of shocks given during CPR and the outcome of CPR in those with a shockable rhythm. The mean number of shocks in the 28 patients who had a shockable rhythm and were discharged alive was 2.21 (95% CI 0.95-3.48). However, the mean number of shocks in the 143 patients who had a shockable rhythm and passed away was 3.91 (95% CI 3.35-4.47), as in Table 3.

**Table 3:** A comparison between the cardiopulmonary resuscitation (CPR) outcome and the number of shocks given *p-value represents the significance of the Mann-Whitney U test N: number of patients

Parameter	Shockable rhythm and discharged alive (N = 28)	Shockable rhythm and passed away (N = 143)	Significance
Mean	2.21	3.91	0.0044*
95% CI	0.95-3.48	3.35-4.47	
Standard deviation	1.91	3.59	

**Figure 4:**
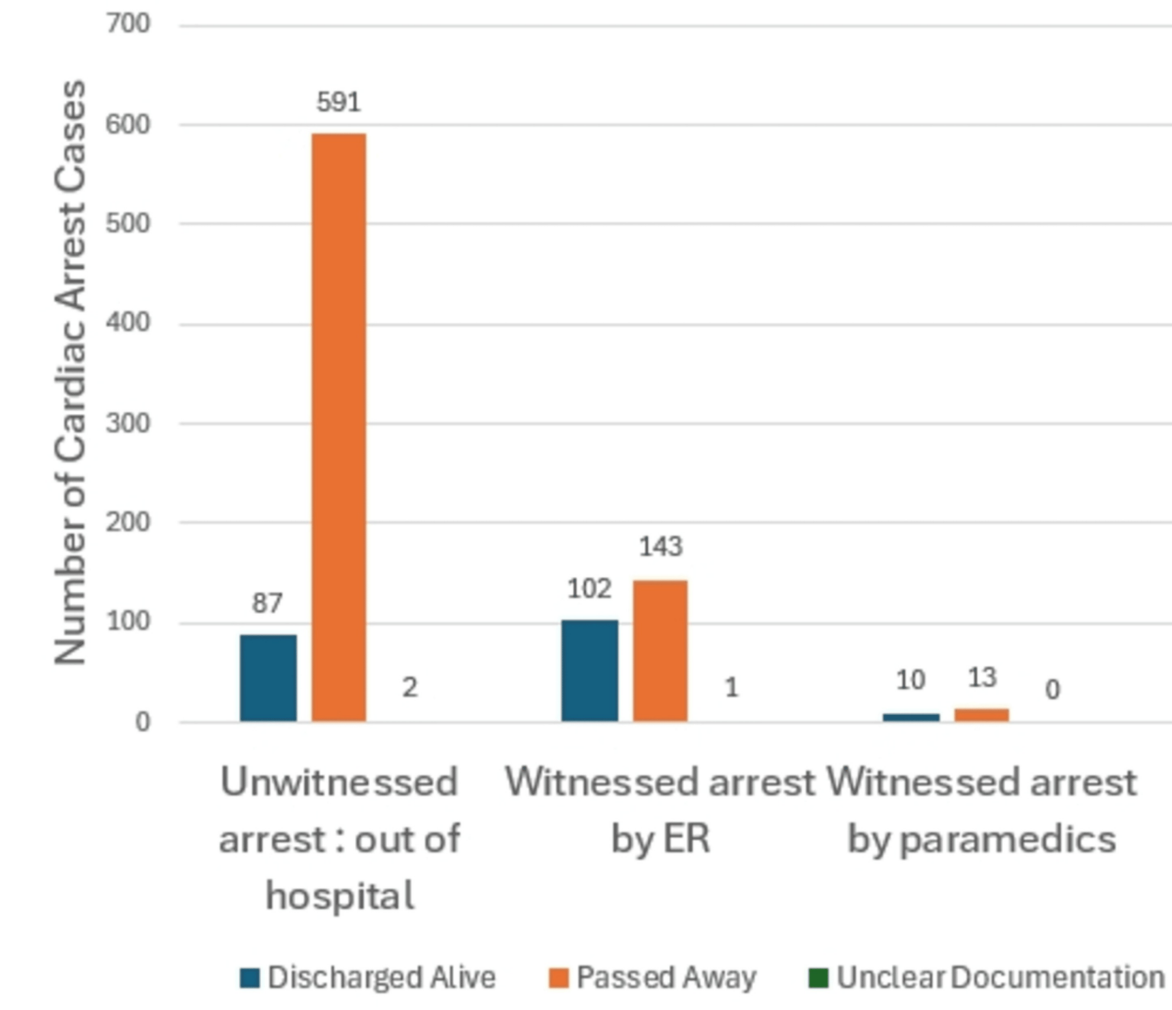
A comparison between the cardiopulmonary resuscitation (CPR) outcome and the setting of occurrence of cardiac arrest p-value < 0.0001 The p-value represents the significance of the chi-square test.

There was a significant difference between the setting of the cardiac arrest before CPR and the outcome of the CPR. Of the 680 patients who had an unwitnessed cardiac arrest, only 87 were discharged alive (12.8%). However, the number of patients who were discharged alive in witnessed arrests in the ED and witnessed arrests by paramedics was 102 out of 246 (41.6%) and 10 out of 23 (43.5%), respectively, as depicted in Figure 4.

There was a significant difference between the initial rhythm of the cardiac arrest and the outcome of the CPR. Four hundred ninety-eight patients had asystole as the initial rhythm of cardiac arrest, of which 52 (10.5%) were discharged alive. Similarly, 16 out of 77 (17.2%) cases of ventricular fibrillation and 4 out of 11 (36.4%) cases of ventricular tachycardia had been discharged alive. Moreover, 122 out of 336 (36.3%) cases of PEA had been discharged alive, as depicted in Table 4.

**Table 4:** A comparison between the CPR outcome and the initial rhythm before starting CPR *p-value represents the significance of the Mann-Whitney U test CPR: cardiopulmonary resuscitation

Initial rhythm	Patients who were discharged alive	Patients who passed away	Significance
Asystole (N = 498)	52 (10.5%)	446 (89.5%)	<0.0001*
Pulseless electrical activity (N = 336)	122 (36.3%)	214 (63.7%)	
Ventricular fibrillation (N = 93)	16 (17.2%)	77 (82.8%)	
Ventricular tachycardia (N = 11)	4 (36.4%)	7 (63.6%)	
Other (N = 11)	5 (45.5%)	6 (54.5%)	

There was no significant difference between the CPR outcome and the time it took the ambulance to come home. Similarly, there was no significant difference between the CPR outcome and the number of minutes the patient stayed in the ambulance.

## Discussion

Relation of duration and time of initiation to the outcome of CPR

The duration of CPR appears to be one of the most significant predictors of the outcome, which is clearly and precisely documented, as it appears that the shorter the duration of the CPR, the better the outcome. Other studies also showed that witnessed and shorter-duration CPR cases were associated with higher survival rates [5]. As mentioned before, the time till initiation also appears to greatly affect the outcome in patients. The greater the delay in initiating CPR, the less likely the patient will survive. There are studies with observational evidence from the prehospital setting that show lower survival rates with greater lengths of time before initiating CPR [2]. This research's findings align with others' conclusions regarding the duration of CPR and the time till initiation of CPR and expected outcomes.

Complications of CPR

CPR is life-saving but at times associated with several complications, mainly due to the nature of the resuscitative efforts. The three main complications observed in our study were IV cannula failure, intubation failure, and rib fractures. Failure rates in the emergency setting are relatively common, as a 10-40% failure rate is described in the literature [10]. These rates could point us toward depending on alternative routes of medication administration, such as intraosseous access. Another main complication observed, intubation failure, is also fairly common, as first-pass success rates are reported to range between 59% and 98% [11]. With this in mind, steps should be taken to explore further and implement supportive measures to enhance intubation success, such as optimizing patient positioning, effectively managing bloody airways, and providing training to improve first-pass intubation rates. The last main complication, rib fractures, has been reported in the literature to be the most common CPR-related injury, with prevalence reported to be 55% among patients undergoing CPR [12]. This highlights the need for providers to be mindful of CPR-related injuries, especially rib fractures, and reduce risk when possible.

Demographics

The majority of patients in this study (90.3%) were from the Riyadh region. This can be explained since Riyadh is the capital city of Saudi Arabia and thus has a higher population than Jeddah and Al-Ahsa.

Out of the 949 patients with cardiac arrest, 672 (70.8%) were men and 277 (29.2%) were women. In this study, 25.7% of female patients were discharged alive, while only 19.1% of males were discharged alive. This finding is in opposition to the findings of other studies, where females were usually the gender with a lower survival rate [13,14]. One study found that while most patients experiencing cardiac arrest were men (62.6%), women (37.4%) who were resuscitated after cardiac arrest had lower survival rates from hospital admission to discharge [13].

In another study, it was observed that women were less likely than men to receive bystander resuscitation. Moreover, when women did receive resuscitation, their survival rates were lower compared to men, a disparity partially attributed to differences in resuscitation characteristics, particularly a lower proportion of shockable initial rhythms [14].

The setting where cardiac arrests occur is crucial as it significantly impacts the outcome. In this study, 71.7% (680 patients) had an unwitnessed cardiac arrest, 25.9% (246 patients) occurred in the ED and were witnessed, and only a small proportion of 2.4% (23 cases) were witnessed by paramedics. Unwitnessed cardiac arrests were linked with poorer outcomes. This is in line with another study, which found that arrests that were not witnessed had a significantly lower likelihood of ROSC and discharge survival [15]. This could be explained because unwitnessed arrests have delayed the start of CPR.

On the other hand, the witnessed arrests were associated with better survival since the treatment was initiated immediately. The survival rate of witnessed cardiac arrests was reported to be 2.81 times higher than that of unwitnessed cases [16].

Rhythm in cardiopulmonary resuscitation

Initial rhythm is a factor that needs to be looked at in a more detailed manner. In our study, only 28 patients out of 171 with shockable rhythm survived, which is a small number given that there is evidence that the survival rate has increased recently. For example, a study conducted over the span of 30 years in Sweden showed an increase in shockable rhythm survival, with a documented increase in overall survival from 14.4% in 1990 to 35.8% in 2020. This could be explained by the rapid improvement in the protocols as the patients started to receive CPR and critical time interventions such as percutaneous coronary intervention more and in a timely manner [17]. This could be one of the reasons shockable rhythm had such a low survival chance, as a delay in invention, such as a defibrillation, could be a contributing factor. Another discrepancy between this study and others is regarding the initial rhythm with the highest probability of survival. In this study, patients who had pulseless electrical activity (36.5%) had the highest percentage of being discharged alive. The “other” category is not representative of survival despite having the highest percentage, as it does not describe one specific rhythm. Contrary to our findings, other studies showed that ventricular tachycardia or ventricular fibrillation was the initial rhythm with the highest probability of survival [8].

Procedures

Seven hundred forty-five cases of CPR had information regarding intubation; only 13.7% were intubated before the start of CPR, 40.5% were intubated during the resuscitation process, and 45.8% did not require intubation. Moreover, data from a national registry of over 150,000 in-hospital cardiac arrests across more than 650 hospitals found that endotracheal intubation was utilized in about 70% of cardiac arrest cases. However, the intubation rate across hospitals varied widely from 27% to 100%, and in patients with no respiratory failure, lower intubation rates were associated with higher survival [18].

Previous studies on cardiac arrest have shown that reducing intubation during CPR may improve the outcome [19-22]. For instance, a comparison of different resuscitation methods that reduced intubation showed better outcomes in terms of survival in out-of-hospital cardiac arrests [19-21]. Delayed intubation has especially been looked at in situations where the cardiac arrest was cardiac in origin with a shockable rhythm; in these cases, limiting intubation and concentrating on chest compressions may improve survival [19-20]. On the other hand, in patients with clinical symptoms of respiratory failure before cardiac arrest, the survival rate was not affected by intubation [22]. This means that intubation may be more beneficial to patients with problems that are respiratory in origin rather than cardiac. This result suggests that there is an incentive to investigate an approach that minimizes intubation in patients with an arrest of cardiac origin.

Limitation

One of the limitations of this research is the study design. Retrospective studies may lead to confounding factors that can be avoided by utilizing prospective studies. Therefore, future studies on the topic of cardiac arrest and CPR are best to be conducted prospectively. Also, the sampling technique used in this study was non-probability consecutive sampling. Thus, the authors recommend employing a randomized probability sampling technique in further studies. Although this study excluded some factors, such as DNR, some factors may also contribute to the patient's morbidity and mortality in the form of confounding factors, such as using a pacemaker or an implantable cardioverter-defibrillator. Another area of improvement for further literature is the methodology part. We suggest applying multivariable analysis represented by an adjusted odds ratio to limit the effect of the confounding factors. Lastly, even though the large sample size can be a strength utilized in this study, it raises the chances of uncovering statistically significant results irrespective of their clinical relevance in the topic of cardiac arrest. Moreover, the sample size of this study, although large, is not sufficient to draw definitive conclusions; further local and international research is necessary to study the variables that affect the efficacy of CPR in cardiac arrest cases and to investigate the variables that increase or decrease the survival rate of patients.

## Conclusions

There were multiple significant findings in this study, most notably that the time to initiation of CPR and the setting in which CPR occurs significantly affect the survival of patients. Most findings are consistent with those available in the literature with some minor differences. Unwitnessed cardiac arrests unfortunately remain to be a challenge with poor survival outcomes. More large multicenter studies are required to draw definitive conclusions regarding the factors that affect survival to then create protocols that can improve in-hospital resuscitative measures and create strategies to better the outcomes of out-of-hospital unwitnessed occurrences.
